# Stage-specific and cell type-specific requirements of *ikzf1* during haematopoietic differentiation in zebrafish

**DOI:** 10.1038/s41598-022-25978-6

**Published:** 2022-12-10

**Authors:** Isabell Hess, Sagar, Connor O´Meara, Dominic Grün, Michael Schorpp, Thomas Boehm

**Affiliations:** 1 Department of Developmental Immunology, Max Planck-Institute of Immunobiology and Epigenetics, Stuebeweg 51, 79108 Freiburg, Germany; 2 Quantitative Single Cell Biology Group, Max Planck-Institute of Immunobiology and Epigenetics, Stuebeweg 51, 79108 Freiburg, Germany; 3 Department of Medicine II, University Hospital Freiburg, Hugstetter Str. 55, 79106 Freiburg, Germany; 4 Würzburg Institute of Systems Immunology, Max Planck Research Group at the Julius-Maximilians-Universität Würzburg, Versbacher Str. 9, 97078 Würzburg, Germany; 5 Helmholtz Centre for Infection Research (HZI), Helmholtz Institute for RNA-Based Infection Research (HIRI), Josef-Schneider-Straße 2, 97080 Würzburg, Germany; 6 Faculty of Medicine, University of Freiburg, Breisacher Str. 153, 79110 Freiburg, Germany

**Keywords:** Developmental biology, Evolution, Genetics, Immunology

## Abstract

The zinc finger transcription factor Ikaros1 (Ikzf1) is required for lymphoid development in mammals. Four zinc fingers constitute its DNA binding domain and two zinc fingers are present in the C-terminal protein interaction module. We describe the phenotypes of zebrafish homozygous for two distinct mutant *ikzf1* alleles. The IT325 variant lacks the C-terminal two zinc fingers, whereas the fr105 variant retains only the first zinc finger of the DNA binding domain. An intact *ikzf1* gene is required for larval T cell development, whereas low levels of adult lymphoid development recover in the mutants. By contrast, the mutants exhibit a signature of increased myelopoiesis at larval and adult stages. Both mutations stimulate erythroid differentiation in larvae, indicating that the C-terminal zinc fingers negatively regulate the extent of red blood cell production. An unexpected differential effect of the two mutants on adult erythropoiesis suggests a direct requirement of an intact DNA binding domain for entry of progenitors into the red blood cell lineage. Collectively, our results reinforce the biological differences between larval and adult haematopoiesis, indicate a stage-specific function of *ikzf1* in regulating the hierarchical bifurcations of differentiation, and assign distinct functions to the DNA binding domain and the C-terminal zinc fingers.

## Introduction

Zebrafish has emerged as a valuable model to examine vertebrate haematopoiesis^1–5^. Yet, much remains to be learned about the genetic regulators of blood cell development in teleosts in order to establish the similarities with and differences to the mammalian system. A major advantage of the zebrafish model is the ease with which genetic screens can be conducted; for instance, forward genetic approaches^6,7^ have resulted in the identification of dozens of genes encoding potential regulators of lymphoid development, including *ikzf1*^6–16^.

Ikzf1 is the founding member of the Ikaros zinc finger family of transcription factors, which consists of five proteins that share a high degree of amino acid similarity^17,18^. In the mouse model, the functions of individual Ikaros family members during various stages of lymphoid development have been amply documented^19^; interestingly, recent work indicated that at least some of these functions are shared by their human counterparts^20–26^. Ikzf1 is thought to prime the lymphoid transcriptional programme in haematopoietic stem cells (HSCs) and to concomittantly repress the transcriptional programmes characteristic of progenitor and non-lymphoid haematopoietic lineages downstream of HSCs^27^. In general, Ikaros-like proteins possess six zinc finger (ZF) motifs; four N-terminal ZFs (one family member, Pegasus, has only three ZFs in this domain) make up the DNA binding module, whereas the two C-terminal ZFs are required for protein–protein interactions^18^. Protein complexes involving Ikaros family members are characterized by homo- and heterotypic interactions, but also extend to interactions with non-family members, explaining the multi-faceted functions of these transcription factors in the positive and negative regulation of their target genes^18^.

In our previous studies, we have characterized a mutant zebrafish *ikzf1* gene encoding a variant protein lacking the lacking the C-terminal ZFs^9^. The mutant fish are characterized by failing larval T cell development; although it later recovers, only few cells complete the maturation process, associated with homeostatic proliferation of T cell clones in the periphery^9,28^. The maturation of B cells is likewise affected, since oligoclonal immature igμ^−^ B cells outnumber igμ^+^ cells^9^. Collectively, our initial studies highlight the important role of the C-terminal ZFs for proper lymphoid development and indicate evolutionarily conserved functions of Ikzf1 in vertebrate haematopoiesis. In a recent study, Huang et al.^29^ examined the larval phenotype of an *ikzf1* allele lacking both DNA binding and interaction domains. Their results defined two critical downstream target genes of Ikzf1, namely *ccr9a*, and *irf4a*; whereas the former is required for homing of haematopoietic progenitors to the thymus, the latter is required to initiate intrathymic T cell differentiation^29^.

Nonetheless, several important questions related to *ikzf1* function remain unanswered, particularly with respect to differences between larval and adult haematopoiesis and its role in regulating the bifurcations of erythro-myeloid and lymphoid differentiation. Here, we address some of these questions by comparing the phenotypes of two *ikzf1* mutants, one lacking both DNA binding and protein interaction modules, the other lacking the protein interaction modules only.

## Results

### Characterization of a new *ikzf1* allele and its role in larval haematopoietic development

**Figure 1:**
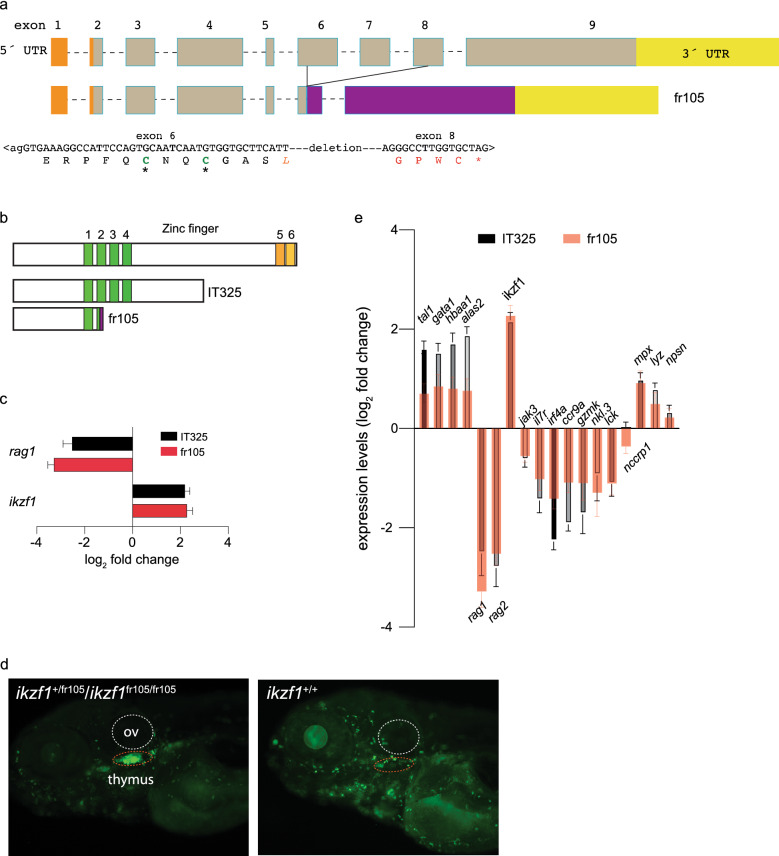
Characterization of a new *ikzf1* allele. (**a**) Schematic structure of the zebrafish *ikzf1* gene. Exons are represented by boxes and numbered and drawn to scale; the introns are indicated by dashed lines but are not drawn to scale. The 5´-UTR and 3´-UTR are indicated in orange and yellow. The CRISPR/Cas9-induced deletion in the fr105 allele is indicated; the nucleotide sequence across the junction is shown and the resulting protein sequence is given in single letter code. The deletion creates a frame-shift, resulting in the addition of unrelated amino acid residues (red letters) before a premature stop codon (*). (**b**) Schematic of the proteins encoded by the two *ikzf1* alleles studied here; the IT325 allele was described by Schorpp et al.^9^. (**c**) Lack of larval T cell development in homozygous IT325 and fr105 mutants as determined by RNA-seq at 5 dpf; *rag1* and *ikzf1* expression levels relative to wildtype are shown (mean ± s.e.m.; n = 3 biological replicas). (**d**) Analysis of the *ikzf1*^*fr105*^ allele. In the *ikaros:eGFP* background, wild-type and heterozygous fish cannot be phenotypically distinguished (left panel), whereas the mutants (right panel) lack fluorescent signals in the thymus (dashed red oval), and exhibit brighter fluorescence. ov, otic vesicle. **e** Differential gene expression patterns in IT325 and fr105 mutants at 5 dpf. The log_2_ fold changes of expression levels relative to wild-type siblings are indicated (mean ± s.e.m.; n = 3 biological replicas).

In our large-scale forward genetic screens^6,7^, we have identified two ENU-induced non-sense recessive mutations in *ikzf1* (ENSDARG00000013539); the variant proteins encoded by mutant alleles II032 (Ref.^6^) and IT325 (Ref.^9^) lack all or part of the two C-terminal zinc-fingers, but retain the four zinc fingers known to be important for DNA binding^18^. Both mutants cause impaired development of lymphoid lineages in zebrafish larvae. In order to examine the role of *ikzf1* in haematopoietic differentiation in more detail and in a comparative fashion, we sought to include in our studies a mutant allele lacking an intact DNA binding domain. To this end, we used the CRISPR/Cas9 system to introduce a deletion in the *ikzf1* gene across the exons encoding the N-terminal zinc fingers. In the fr105 allele (*ikzf1*^*fr105*^), an intragenic deletion removes part of exon 6, the entirety of exon 7, and part of exon 8 (c.del513-819, p.Phe171LeuX4; ENSDART00000016430.7) (Fig. 1a), leading to a truncated Ikzf1 protein, retaining only zinc finger 1 (Fig. 1b). As expected from studies of the IT325 allele^9,28^, fish homozygous for the fr105 allele lack larval T cell development, as indicated by the more than tenfold reduction of *rag1*-transcripts observed in RNA-seq at 5 days after fertilization (5dpf) (Fig. 1c; Supplementary Data 1 and 2). Collectively, our results indicate that the early stages of larval lymphoid development are drastically impaired in fr105 mutants, compatible with a previous analysis of a similar *ikzf1* mutant that lacks all six zinc fingers^29^. We conclude that larval lymphoid development is dependent on an intact *ikzf1* gene^9,28^. Unexpectedly, however, we found that in mutants homozygous for both IT325 and fr105 alleles, the levels of the *ikzf1* gene are significantly upregulated (Fig. 1c), indicating that the Ikzf1 transcription factor is part of a negative feedback loop regulating its own expression levels. This result is confirmed by analysis of fish carrying an *ikzf1:eGFP* reporter transgene^28^; in the absence of an intact *ikzf1* gene, GFP expression levels are much higher than in *ikzf1*-sufficient siblings (Fig. 1d). This genetic constellation also proved useful in establishing a genotype-phenotype correlation in intercrosses of *ikzf1*^+/fr105^ heterozygous parents. Whereas the genotypes segregate according to Mendelian ratios, the phenotypes segregate in a 3:1 pattern, both with respect to the size of the thymus and the expression levels of GFP (Fig. 1d), indicating that the fr105 allele is recessive.

Given the expression of *ikzf1* in several haematopoietic lineages^18^, we performed RNA-seq on 5 dpf larvae of IT325 and fr105 mutants and examined the expression of genes indicative of lymphoid and non-lymphoid lineages in IT325 and fr105 mutants. As expected, genes indicative of larval T cell development (*rag1*, *rag2*, *jak3*, and *il7r*) are expressed at lower levels in both mutants. The same difference is found for the *irf4a*-*ccr9a* axis^30^; the expression of other genes associated with lymphoid differentiation (*lck*, *gzmk*, *nkl.3*, *nccrp1*) is also reduced (Fig. 1e). Interestingly, the IT325 mutant protein, which preserves the DNA binding domain but lacks the two C-terminal zinc fingers, has a more detrimental effect on the expression of most genes, presumably because this variant acts in a dominant negative fashion. Whereas lymphoid development is impaired in the absence of an intact *ikzf1* gene, we unexpectedly found that genes associated with the differentiation of the erythroid and myeloid lineages are upregulated in our mutant fish. For instance, the erythroid-lineage genes *tal1*, *gata1a*, *alas2*, and *hbaa1* are coordinately upregulated in both mutants, although the magnitude of increase is four times higher in IT325 mutants than it is in fr105 mutants (Fig. 1e). Analysis of upregulated myeloid lineage-related genes (*mpx*, *lyz*, and *nspn*) also reveals allele-specific differential effects (Fig. 1e). Collectively, non-lymphoid genes are expressed at higher levels in mutant larvae, compatible with the notion that Ikzf1 suppresses non-lymphoid cell fates^18^. Supplementary Data 1 and 2 tabulate the differentially expressed genes in 5 dpf mutant larvae homozygous for the IT325 and fr105 alleles, respectively. To provide a comprehensive view on the functional consequences of the transcriptional changes in the mutants, we carried out Panther^31,32^ and Reactome^33^ pathway analyses for upregulated and downregulated genes respectively; Table 1 lists the significantly enriched pathways related to haematopoietic differentiation, whereas Supplementary Data 3 records the full analysis. Apart from the alterations of haematopoiesis-related pathways, changes in neuronal signalling/development are found, compatible with the activity of Ikzf1 in a subset of neurons^34,35^.

**Table 1:** Differentially regulated haematopoietic pathways in *ikzf1* mutants.

	Fold Enrichment	FDR
**Upregulated genes in 5 dpf IT325 mutants**		
Heme biosynthesis (P02746)	5.04	2.93E-02
Ubiquitin proteasome pathway (P00060)	2.83	1.16E-02
**Downregulated genes in 5 dpf IT325 mutants**		
B cell activation	3.98	5.82E-08
T cell activation (P00053)	3.47	9.52E-08
Apoptosis signaling pathway (P00006)	3.13	5.72E-08
Inflammation mediated by chemokine and cytokine signaling pathway (P00031)	2.28	6.36E-08
**Upregulated genes in 5 dpf fr105 mutants**		
Heme biosynthesis (P02746)	6.34	9.43E-03
DNA replication (P00017)	4.59	4.23E-03
Integrin signalling pathway (P00034)	2.07	3.23E-03
**Downregulated genes in 5 dpf fr105 mutants**		
B cell activation (P00010)	4.27	8.05E-07
T cell activation (P00053)	3.3	4.53E-05
Apoptosis signaling pathway (P00006)	3.05	1.47E-05
Inflammation mediated by chemokine and cytokine signaling pathway (P00031)	2.52	2.49E-07

### Haematopoietic differentiation in the kidney marrow of adult mutants

**Figure 2:**
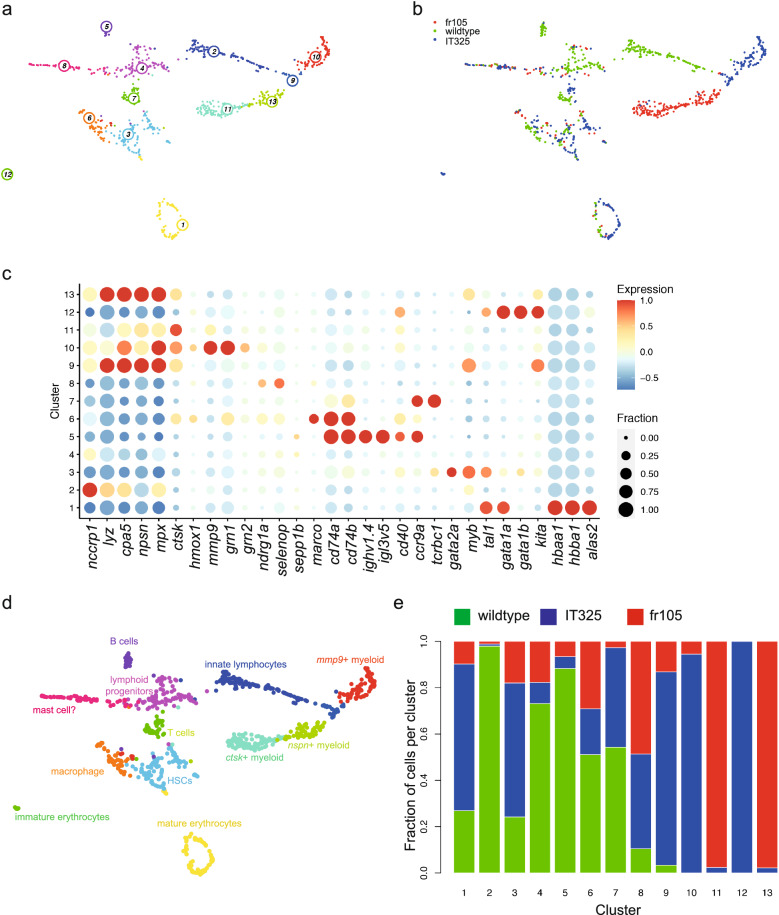
Transcriptional landscape of whole kidney marrow cells of IT325 and fr105 mutants. (**a**) Uniform manifold approximation and projection (UMAP) representation of transcriptome similarities determined by combined analysis of 341 wild-type, 331 mutant cells of the IT325 line, and 262 mutant cells of the fr105 line; the individual clusters are indicated by colour and numbers. Fish were 3 months of age. (**b**) Distribution of wild-type and mutant cells among transcriptionally defined cell clusters; origins of cells are colour-coded. (**c**) Expression pattern of signature genes (listed at the bottom) in the individual cell clusters shown in (**a**, **b**). Colour represents the z-score of the mean expression of the gene in the respective cluster and dot size represents the fraction of cells in the cluster expressing the gene. z-scores above 1 and below -1 are replaced by 1 and −1, respectively. (**d**) Cell type identification based on signature gene expression patterns (see Table 2, and text for details). (**e**) Distribution of cells of different genotypes in the identified cell clusters.

The perturbations of larval haematopoiesis as revealed by bulk RNA-seq analysis could be explained by dysregulation of genes and/or altered compositions of cell types in the haematopoietic compartment. In order to examine this in more detail, and to determine whether the alterations persist in adult fish, we used scRNA-seq by mCEL-Seq2 (Ref.^36,37^) (Supplementary Fig. 1) to examine the cellular heterogeneity of whole kidney marrow (WKM) cells in wild-type and mutant fish. Cells with similar transcriptional profiles were identified by Louvain clustering using VarID^38^; the resulting UMAP representations^39^ illustrate the complexity of the adult haematopoietic compartment in terms of gene expression profiles and cluster sizes (Fig. 2a,b). The combined analysis of WKM cells of wild-type and the two mutants resulted in 13 distinct transcriptionally defined clusters (Fig. 2a,b). We compiled lists of the differentially expressed genes in each of these clusters (Supplementary Data 4) and used a manually curated selection of these genes to generate a diagnostic matrix of gene expression in the transcriptionally defined cell clusters (Fig. 2c; Table 2).

**Table 2:** Gene expression profiles of haematopoietic lineages identified by scRNA of WKM marrow cells.

Lineage	Diagnostic gene profile	Cluster ID (Fig. 2d)	Cluster ID (Fig. 3d)	Cluster ID (Fig. 4d)
HSC	*myb, tal1, gata2a*	3	4	
immature T cell	*myb, tcrbc1, ccr9a*		11	
T cell	*tcrbc1, ccr9a*	7	6	7
B cell	*igh, igl, cd40, ccr9a, cd74a, cd74b*	5	2	6
innate lymphocyte	*nccrp1*	2	1	4
mature erythrocytes	*hbaa1, hbba1, alas2*	1	5	1
immature erythrocytes	*tal1, gata1a, gata1b*	12	7,8	
immature myeloid cells	*myb, mpx, nspn, kita, cpa5, lyz*	9	12	2, 10
nspn+ myeloid cells	*mpx, npsn, lyz*	13		
mmp9+ myeloid cells	*mpx, mmp9, cpa5, grn1*	10	10	11
ctsk+ myeloid cells	*ctsk*	11		11
macrophage	*cd74a, cd74b, marco*	6	3	3

For instance, in the lymphoid lineage, a cluster of T cells (cluster 7) was identified by the co-expression of the T cell receptor gene (*tcrbc1*) and the *ccr9a* gene encoding a lymphoid-specific chemokine receptor. B cells were identified by the expression of immunoglobulin heavy chain (*ighv1.4*) and light chain (*igl3v5*) genes in addition to the genes encoding the co-stimulatory molecule Cd40 and the chemokine receptor Ccr9a (cluster 5). High expression levels of genes (*cd74a*; *cd74b*) associated with the MHC class II pathway are not only found on B cells, but also on cells in cluster 6; expression of the *marco* gene, encoding a scavenger receptor, suggests that cells in this cluster are macrophages^40^. Cells belonging to the erythroid lineage are found in clusters 1, and 12. Mature erythrocytes (cluster 1) express high levels of haemoglobin genes (here exemplified by *hbaa1*, and *hbba1*) and other erythrocyte-specific genes, such as *alas2,* encoding 5'-aminolevulinate synthase 2 important for the regulation of iron metabolism^41^; more immature cells of the erythroid lineage (cluster 12) express high levels of *tal1*, *gata1a* and *gata1b*, encoding key transcription factors of this lineage^7,42^. Haematopoietic progenitor cells, expressing the stem-cell associated genes for the transcription factors Myb, Tal1, and Gata2a, are identified in cluster 3.

Several cell clusters are associated with various types of myeloid cells. For instance, cells expressing high levels of *mpx*, encoding myeloperoxidase, are predominantly found in clusters 9, 10, and 13. Based on the co-expression profiles, we suggest that cluster 9 harbours more immature myeloid cells (presence of significant levels of *myb*), whereas cluster 13 likely represents neutrophils, which exhibit high levels of *mpx*^43^, *npsn*, a gene encoding a metalloprotease involved in the host defense against bacterial infection^44^, and *lyz*, encoding lysozyme, a glycoside hydrolase expressed exclusively in myeloid cells and also involved in anti-bacterial defense^45^. Clusters 10 and 11 are populated by *mmp9*^+^ (cluster 10), and *ctsk*^+^ (cluster 11) neutrophil-like cells, an assignment that is supported by their close affinity to immature myeloid cells (cluster 9) in the UMAP representation (Fig. 2a). Innate lymphocyte-like cells are grouped together in cluster 2, which exhibits high levels of the *nccrp1* gene, encoding the non-specific cytotoxic cell receptor protein 1 homolog^46^. Based on our diagnostic expression paradigm (Table 2), cells in clusters 4 and 8 lack unambiguous lineage expression patterns, although they can be distinguished by the expression of *ndrg1a* and *seleop* genes. To explore their potential identity further, we subjected the list of enriched genes to pathway analysis. Neither Panther nor Reactome algorithms gave a hint as to their cellular identity; however, for cluster 4, GO analysis^47^ returned a single significant hit for long-chain fatty acid biosynthetic process (GO:0042759; adjusted *P* = 0.020). Given that cluster 4 is dominated by wildtype cells (see below) and positioned between B cells (cluster 5) and T cells (cluster 7), it is possible that cells in cluster 4 represent immature lymphoid cells. The co-expression of *ndrg1a*, *seleop*, and *kita* suggests that that cells in cluster 8 may represent mast cell lineage precursors. A summary of cluster assignments projected onto the UMAP is presented in Fig. 2d (see also Table 2).

Interestingly, cells of the three genotypes contribute differentially to the 13 cell clusters identified by the combined analysis. For instance, the B cell cluster (cluster 5) is dominated by wild-type cells, whereas cells assigned to the population of immature erythrocytes (cluster 12) consists primarily of cells derived from the IT325 mutant (Fig. 2e). In total, 8/13 cell clusters are dominated by mutant cells, although the relative contributions of the two mutants also varies from cluster to cluster (Fig. 2e). These observations indicate that the impact on haematopoietic differentiation of the two mutants substantially differs.

### One-to-one comparisons of *ikzf1* mutants with wild-type fish

**Figure 3:**
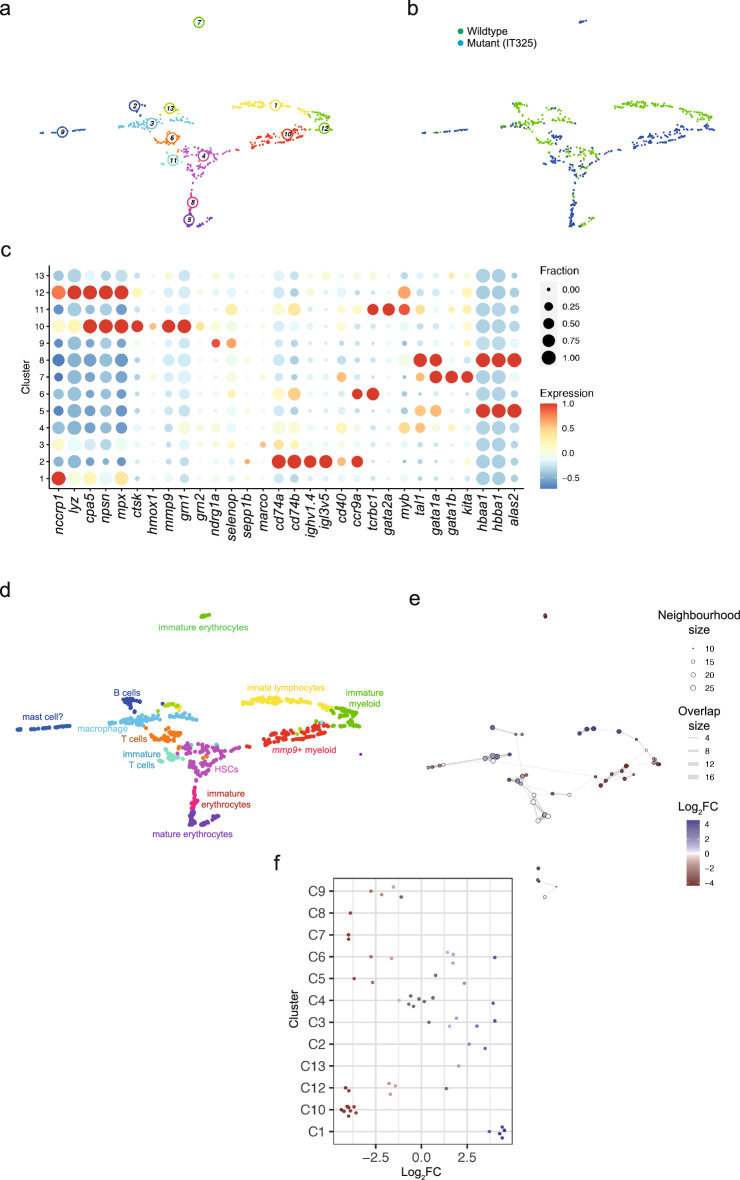
Transcriptional landscape of whole kidney marrow cells of IT325 mutants. (**a**) Uniform manifold approximation and projection (UMAP) representation of transcriptome similarities determined by combined analysis of 341 wild-type and 331 mutant cells of the IT325 line; the individual clusters are indicated by colour and numbers. (**b**) Distribution of wild-type and mutant cells among transcriptionally defined cell clusters; genotypes of cells are colour-coded. (**c**) Expression pattern of signature genes (listed at the bottom) in the individual cell clusters shown in (**a**, **b**). Colour represents the z-score of the mean expression of the gene in the respective cluster and dot size represents the fraction of cells in the cluster expressing the gene. z-scores above 1 and below -1 are replaced by 1 and −1, respectively. (**d**) Cell type identification based on signature gene expression patterns (see Table 2, and text for details). (**e**) Graph representation of neighbourhoods identified by the Milo algorithm; the positions of index cells of neighbourhoods are projected onto their positions of the UMAP representation shown in (**a**, **b**). Nodes are equivalent to neighbourhoods; colours indicate their compositional differences between wildtype and mutant cells and are quantified as log_2_ fold changes at FDR 10% (colour code is indicated to the right); the sizes of the nodes denote the number of cells in the neighbourhood; graph edges represent the number of cells shared among adjacent neighbourhoods. (**f**) The distribution of log_2_ fold changes in abundance between wildtype and mutant cells in neighbourhoods identified in different cell clusters (identified at the left) is shown in a bee-swarm plot; neighbourhoods exhibiting differential abundance at FDR 10% are colour-coded (reddish colours denote greater abundance of mutant cells; bluish colours denote greater abundance of wildtype cells).

**Figure 4:**
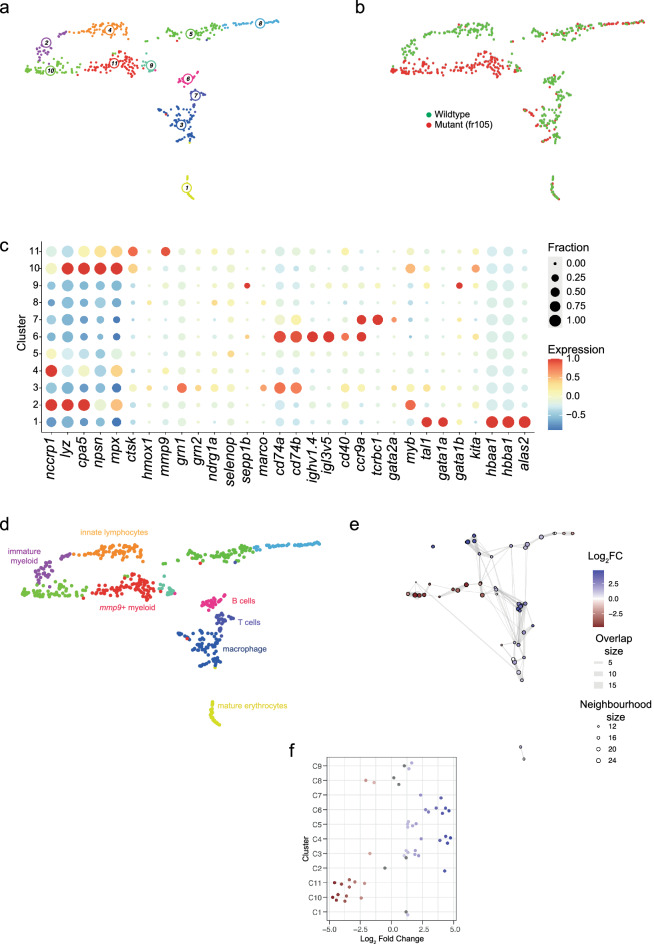
Transcriptional landscape of whole kidney marrow cells of fr105 mutants. (**a**) Uniform manifold approximation and projection (UMAP) representation of transcriptome similarities determined by combined analysis of 341 wild-type and 262 mutant cells of the fr105 line; the individual clusters are indicated by colour and numbers. (**b**) Distribution of wild-type and mutant cells among transcriptionally defined cell clusters; origins of cells are colour-coded. (**c**) Expression pattern of signature genes (listed at the bottom) in the individual cell clusters shown in (**a**, **b**). Colour represents the z-score of the mean expression of the gene in the respective cluster and dot size represents the fraction of cells in the cluster expressing the gene. z-scores above 1 and below -1 are replaced by 1 and −1, respectively. (**d**) Cell type identification based on signature gene expression patterns (see Table 2, and text for details). (**e**) Graph representation of neighbourhoods identified by the Milo algorithm; see Fig. 3e legend for explanation. (**f**) Bee-swarm plot of differential abundance analysis; see Fig. 3f legend for further explanation.

Next**,** we proceeded to disentangle the effects of the two mutants by performing individual comparisons of the two mutants with wild-type cells. In this section, we give a brief overview of these analyses, before individually discussing the characteristics of the two mutants in the following sections. The global patterns of differential gene expression are detailed in Supplementary Data 5 (IT 325 mutant) and Supplementary Data 6 (fr105 mutant), and the results of subsequent pathway analyses are summarized in Supplementary Data 7. For the fine-grained analysis, we calculated the UMAP projections after VarID clustering (Fig. 3 [IT325 mutant] and Fig. 4 [fr105 mutant]), and then determined the expression levels of differentially expressed genes in each cluster of the two analyses (Supplementary Data 8 [IT325 mutant] and Supplementary Data 9 [fr105 mutant]); we then executed pathway analyses for each cluster (Supplementary Data 10 [IT325 mutant] and Supplementary Data 11 [fr105 mutant]) to assist and complement the lineage assignment. In a subsequent step, we analyzed the differential contribution of wild-type and mutant cells by use of the Milo algorithm that tests differential abundance by assigning cells to partially overlapping neighbourhoods on a k-nearest neighbour graph^48^ (Fig. 3e,f [IT325 mutant] and Fig. 4e,f [fr105 mutant]).

The results of the individual comparisons (see the following two sections) highlight substantial differences between the two mutants as exemplified by the bias towards erythroid cell lineage differentiation in the IT325 mutant.

### Perturbations caused by the *ikzf1*^IT325^ mutant lacking the dimerization domains

Comparison of kidney marrow cells of wild-type and IT325 mutants resulted in the identification of 13 clusters (Fig. 3a,b). Differential gene expression analysis (Fig. 3c) identified the major components of the haematopoietic system. T cells are found in clusters 6 and 11, B cells define cluster 2, and erythrocyte-lineage cells are found in clusters 5, 7, and 8. Innate lymphocytes are found in cluster 1, HSCs in cluster 4, and myeloid sub-populations in clusters 3, 9, 10, and 12 (Fig. 3d; Table 2).

We have previously shown that despite failing larval T cell development, T lineage cells can be recovered from adolescent IT325 mutant fish, although most of these cells appeared to be immature^9,28^. The scRNA-seq data presented here confirm and extend this result. Transcriptionally, cluster 6, which contains mature T cells, is closely related to cluster 11, which encompasses immature T cells (Fig. 3a). Interestingly, cluster 11 contains mostly mutant cells (Fig. 3b); the co-expression of *tcrbc1*, *gata2a* and *myb* indicates that these are immature cells, in line with our previous conclusion of a partial block of T cell development in adult IT325 mutants^9^. By contrast, cluster 6 is populated by cells of both wildtype and mutant origin (Fig. 3b). Taken together, this observation indicates the presence of a pronounced T cell maturation block in the IT325 mutant. Cluster 2 is dominated by B cells of wild-type genotype that express high levels of immunoglobulin genes and other maturation markers, indicating that also B cell development is severely blocked in the mutants. Moreover, cells in cluster 1, distinguished by high levels of *nccrp1* expression that is associated with innate NK-like cells, is composed entirely of wild-type cells. Collectively, these results indicate that IT325 mutants suffer from greatly impaired development of the principal lymphoid lineages.

In contrast to the lymphoid compartment, wild-type cells constitute only a minor fraction of the erythroid lineage, indicating that this non-lymphoid differentiation pathway is greatly favoured in the mutant. Indeed, the large number of erythroid lineage cells present in the mutant kidney marrow facilitates the identification of two types of immature cell types (clusters 7 and 8), both of which are dominated by cells of mutant phenotype. Based on the expression patterns of progenitor genes (such as *gata1a* and *gata1b*), we propose that cells in cluster 7 are more immature than those in cluster 8, wherein cells co-express haemoglobin genes. The cluster of mature erythrocytes (cluster 5), contains both wild-type and mutant cells (Fig. 3a–c).

Among myeloid cells, cluster 10 is populated exclusively by mutant cells, whereas another (cluster 12) contains both wild-type and mutant cells of an immature myeloid expression profile, as illustrated by the presence of appreciable levels of *myb*.

The results obtained by clustering using the VarID algorithm are confirmed by neighbourhood analysis using the Milo algorithm (Fig. 3e,f); note that because of the low numbers of cells in cluster 11, they are not assigned to a particular neighbourhood.

In conclusion, our scRNA-seq analysis of adult fishes identifies an unexpected lineage diversion in the IT325 mutant. The partial block of lymphoid development is accompanied by a strong bias towards erythroid differentiation. Thus, it appears that the IT325 mutation manifests itself in a similar lineage bias in larval (Fig. 1) and adult (Fig. 3) haematopoiesis.

### Perturbations caused by the *ikzf1*^fr105^ mutant lacking DNA binding and dimerization domains

Comparison of kidney marrow cells of wild-type and fr105 mutants resulted in the identification of 11 clusters (Fig. 4a,b), for which differential gene expression analysis (Fig. 4c) identified the major components of the haematopoietic system (Fig. 4d; Table 2). Interestingly, in this instance, the gene expression profiles indicate that T cells (cluster 7) and B cells (cluster 6) are of mature phenotype, but all cells originate from wild-type siblings. Innate lymphoid cells expressing *nccrp1* are found in cluster 4, which is dominated by wild-type cells (Fig. 4e,f). In sum, adult lymphopoiesis in fr105 mutants is severely impaired.

In contrast to the situation in IT325 mutants, no evidence for enhanced erythropoiesis is seen for fr105 mutants; in fact, cluster 1, which encompasses cells of the erythroid lineage is dominated by wild-type cells. Thus, the moderately enhanced erythroid differentiation in the larval stage (Fig. 1) does not persist into adulthood.

Among myeloid cells, only two clusters (clusters 10 and 11) are predominantly populated by mutant cells; interestingly, whereas cells in cluster 10 exhibit a transcriptional signature of immature myeloid cells, cluster 11 is composed of cells expressing high levels of *ctsk* and *mmp9*, reminiscent of cluster 10 in the wild-type/IT325 analysis (Fig. 3). In both cases, these clusters are composed almost entirely of mutant cells. We conclude that this unique type of myeloid population, which represents only a minor fraction in wild-type kidney marrow, is strongly favoured in the absence of a wild-type *ikzf1* gene. In sum, the scRNA-seq analysis of adult fr105 mutants (carrying an *ikzf1* allele lacking intact DNA binding and activation domains) indicates a severe block of lymphoid differentiation, noticeably impaired erythropoiesis, and profound alterations of myeloid differentiation.

## Discussion

The first hint that *ikzf1* is involved in zebrafish lymphoid development was provided by the discovery of its expression in haematopietic tissues during zebrafish development^49^. This hypothesis was confirmed by the phenotype of zebrafish lacking an intact *ikzf1* gene^9^. Subsequent studies indicated that Ikzf1 regulates the expression of *irf4a*^29^, which in turn was found to control *ccr9a* expression^30^. Interestingly, at the larval stage, the *ikzf1*-*irf4a* axis appeared to be required to efficiently repress *pu.1* expression to prevent alternate differentiation paths in T lymphoid-primed progenitors^30^. The results presented here support the conclusion that lack of an intact *ikzf1* gene favours myeloid differentiation relative to lymphoid development; these alterations may be mirrored in myeloid dysfunctions in humans carrying mutant IKFZ1 alleles^25,50^. Interestingly, a mouse hypomorphic *Ikzf1* allele that contains DNA binding and dimerization domains is associated with higher neutrophil numbers, owing to a change in the migratory potential and survival of neutrophil precursors^51^, pointing to a critical threshold of Ikzf1 protein function in regulating decisions at the lymphoid/myeloid-erythroid branch point.

Interestingly, our results indicate that the two *ikzf1* mutants analysed here exhibit distinctly different phenotypes in adult haematopoiesis. With respect to T cell development, it appears that the ΔZF2-6 (fr105) mutant blocks differentiation at an earlier stage than the ΔZF5,6 (IT325) mutant, whereas their effect on B cell development and innate cytotoxic lineages are similar. The mutants also behave similarly with respect to the characteristic myeloid-biased differentiation trajectories in larval and adult haematopoiesis. By contrast, the two mutations differ sharply regarding their effects on adult erythropoiesis. The stimulatory effects on larval erythropoiesis are qualitatively similar but more pronounced in the ΔZF5,6 (IT325) mutant. We speculate that the C-terminal interaction module is involved in creating a repressive environment at some of Ikzf1´s target genes^18^; thus, a dominant negative effect of the C-terminally truncated mutant lifts the repression of the erythroid transcriptional landscape. By contrast, adult erythropiesis is severely compromised in the ΔZF2-6 (fr105) mutant, whereas it remains at supraphysiologial levels in the ΔZF5,6 (IT325) variant. This may be explained by distinct functions of the two zinc finger modules of Ikzf1: At the adult stage, its DNA binding activity is essential for efficient erythroid differentiation, and the C-terminal zinc fingers serve to constrain excesssive activity at the expense of other haematopoietic lineages. The functional distinction between these two modules appears to be less clear-cut during larval erythropoiesis, since erythropoiesis still occurs at supraphysiological levels even in the mutant without an intact DNA binding domain; we speculate that other factors can replace Ikzf1 function at this stage, which are less efficient at restricting entry into the erythroid differentiation pathway. The observation that the expression of *gata1* and *be1*-globin genes was unaltered in *irf4a*-deficient embryos^30^ suggests that Ikzf1 regulates erythroid development via a distinct regulatory module than lymphoid development.

Collectively, the present results reinforce the biological differences between larval and adult haematopoiesis, indicate a stage-specific function of ikzf1 in regulating the hierarchical bifurcations of lymphoid and non-lymphoid differentiation trajectories, and assign distinct functions to the DNA binding domain and the C-terminal zinc fingers of Ikzf1.

## Methods

### Animals

Zebrafish (Danio rerio) strain Tüpfel long fin/Ekkwill was maintained in the animal facility of the Max Planck Institute of Immunobiology and Epigenetics and used for the experiments described here.

Ethics declaration. All experimental protocols were approved by the ethics committee of the Max Planck Institute of Immunobiology and Epigenetics, under license permit AZ 35-9185.81/G-14/41 issued by the Regierungspräsidium Freiburg, Germany. All methods and procedures were carried out in accordance with the relevant guidelines and regulations. All methods are reported in accordance with ARRIVE guidelines. The following information is given in the manuscript: genotypes of animals, groups of animals being compared, sample size, outcome measures, statistical methods, and relevant experimental procedures. No animal was excluded from the study, randomization was not performed, and experimenters were not blinded.

### CRISPR mutants

CRISPR guide RNAs (5´-GGTGCTTCATTCACTCAGAA [located in exon 6 of transcript ENSDART00000016430.7]; 5´-GGACATGCCTGCATCTGAGA [located in exon 8 of transcript ENSDART00000016430.7]) were created by incubating overlapping primers of the target sequences (5 μg/primer, 100 mM MgCl_2_, 0.1 M Tris pH 7.5) at 95 °C for 5 min and cooling to RT. Annealed primers were ligated into BsaI-digested pDR274 vector (50 ng annealed primers, 10 ng BsaI-digested pDR274, 5 U T4 ligase, 1X T4 ligase Buffer) for 2 h at 22 °C, with the reaction inactivated at 65 °C for 10 min. The ligation mixtures were dialyzed and transformed into *E.coli* DH5α by electroporation. Culture of transformants, plasmid extraction and in vitro transcription of guide RNAs were carried out as described^52^. Purified CRISPR guide RNAs were tested for specificity by in vitro digestion of target DNA (80 ng PCR amplicon containing target sequence, 600 ng Cas9 protein from *Streptococcus pyogenes* [PNA Bio], 300 ng guide RNA, 1X CutSmart buffer [New England Biolabs]) at 37 °C for 1 h. Guide RNA was removed by adding 4 μg of RNAse A to the reaction for 15 min at 37 °C prior to visualization of cleavage products by agarose gel electrophoresis. The extent of in vitro digestion of target DNA was compared between reactions with and without addition of guide RNA. CRISPRs were titrated and injected in a volume of 1–2 nL into wild-type embryos at the 1-cell stage in a solution containing (250 ng/μL guide RNA, 500 ng/μL Cas9 protein, 1% phenol red, Danieau buffer). Carriers of genetic lesions were outcrossed to wild-type fish, and stable carrier lines were created from suitable mutations. The structure of the fr105 allele is as follows. It caries a deletion of ~ 3.6 kb on chromosome 13 (nucleotides 15962388 to 15966005; GRCz11). Genotyping was carried out as follows. To detect the intragenic deletion, primers CRISPRikarosMS1: 5’-cctttacctatttatgtggagtg and CRISPRikaros MS2: 5’-tgcatattaaacagtgatcgctg (amplicon size 260 bp) were used; the wildtype allele was detected using primers 5’-acgctctcactggacatctg and 5’-tgcactgcaagtagttgtgac (the amplicon is located in the deletion; size 230 bp). During the establishment of the *ikzf1*^*fr105*^ allele, RNA in situ hybridisation with *rag1* and *gh* gene-specific probes was used to identify homozygous mutants at 5 d.p.f. (Ref. 9); in addition, mutants were also identified on an *ikzf1:eGFP* transgenic background^28^. Fish of *ikzf1*^+/+^ and *ikzf1*^+/fr105^ genotypes are indistinguishable based on GFP fluorescence patterns; by contrast, *ikzf*^*fr105*/fr105^ mutants are easily recognizable by the increased GFP fluoresence levels and the absence of thymus staining.

### Flow cytometry of zebrafish cells

Flow cytometric analysis of light-scatter characteristics of WKM cells followed^53^ (see Supplementary Fig. 1); staining with hydroxystilbamidine (Enzo Life Sciences; final concentration 1 μg/mL) was used to exclude dead cells. Cells were sorted using a BD Fortessa II instrument from Dako Cytomation-Beckman Coulter.

### RNA extraction and cDNA synthesis

Zebrafish embryos (5 d.p.f.; 3 individual fish per genotype) were homogenised in 100 μL of Tri Reagent (Sigma, Cat#93,289) and transferred to 2 mL deep 96 well plates containing an additional 400 μL of Tri Reagent. The RNA-containing aqueous phase was stored at −80 °C until genotyping was completed. DNA was extracted from the interphase and organic phase according to the manufacturer’s instructions. Following genotyping, RNA was extracted from homozygous mutants and homozygous wild-type siblings. DNA was removed from RNA extraction using TURBO DNA-free kit (Invitrogen, Cat#AM1907). RNA was quantified using the Qubit RNA HS Assay Kit (ThermoFisherScientific, Cat#Q32852) and the Qubit 4 Fluorometer (ThermoFisherScientific, Q33226). RNA quality was checked by determining the 18S/28S rRNA ratio using the Fragment Analyzer RNA Kit (ThermoScientific, Cat#DNF-471-0500) and the 5200 Fragment Analyzer System (ThermoScientific, Cat#M5310AA). cDNA libraries were prepared from 1 μg of mRNA following poly-A selection using TruSeq stranded mRNA Library Prep (Illumina, Cat#20,020,595) according to manufacturer’s instructions.

### RNA sequencing and computational analysis of RNA-seq data

RNA-Seq was performed using mutant and wild-type siblings from each zebrafish line. The libraries were sequenced in paired-end 75 bp mode at a depth of 25 million reads on an Illumina HiSeq 2500/3000 instrument. Reads were aligned to the reference genome with STAR 2.5.2b-1 (Ref.^54^) and the reference annotation from Ensembl (Zv10.85, http://www.ensembl.org/info/data/ftp/index.html). The resulting alignments were quantified at the gene level with featureCounts version 1.6.0.1 (Ref.^55^) and differential expression performed using DESeq2 version 2.11.40.1 (Ref.^56^). The analysis was orchestrated on the in-house version of the Galaxy server based on the Galaxy platform^57^. All tools were used with default parameters unless otherwise stated.

### Single-cell RNA amplification and library preparation

Single-cell RNA sequencing was performed using the mCEL-Seq2 protocol, an automated and miniaturized version of CEL-Seq2 on a mosquito nanoliter-scale liquid-handling robot (TTP LabTech)^36,37^. WKM cells of 3 month-old wildtype and mutant fish were sorted according to light scatter characteristics (see Supplementary Fig. 1) and processed in parallel to minimize batch effects during subsequent processing steps. Eight libraries with 96 cells each for each genotype were sequenced per lane on Illumina HiSeq 2500 sequencing system (pair-end multiplexing run) at a depth of ~ 130,000–200,000 reads per cell. Sequencing was performed at the sequencing facility of the Max Planck Institute of Immunobiolgy and Epigenetics.

### Quantification of transcript abundance

Paired end reads were aligned to the transcriptome using bwa (version 0.6.2-r126) with default parameters^58^. The right mate of each read pair was mapped to the ensemble of improved zebrafish gene models and to the set of 92 ERCC spike-ins in sense direction^59^. Zebrafish gene models were based on Ensembl release 74 (http://www.ensembl.org) and were improved for 3’ UTR annotations as described previously^60^. Reads mapping to multiple loci were discarded. The left read contains the barcode information: the first six bases corresponded to the unique molecular identifier (UMI) followed by six bases representing the cell specific barcode. The remainder of the left read contains a polyT stretch. For each cell barcode, the number of UMIs per transcript was counted and aggregated across all transcripts derived from the same gene locus. Based on binomial statistics, the number of observed UMIs was converted into transcript counts^61^.

### Single-cell RNA sequencing data analysis

Clustering analysis and visualization of all datasets were performed by the VarID algorithm^38^. Cells with a total number of transcripts < 1500 were discarded and the count data of the remaining cells were normalized by downscaling (Supplementary Fig. 1). The pruned kNN matrix was inferred using the pruneKnn function of VarID with the default parameters except alpha (set to 1) and no_cores (set to 10). For the joint analysis of WT and fr105 mutant, the knn parameter in the pruneKnn function was set to 8. The Uniform manifold approximation and projection for dimension reduction (UMAP) representation was used for cell cluster visualization^39^. Differentially expressed genes between two subgroups of cells were identified similar to a previously published method^62^. First, negative binomial distributions reflecting the gene expression variability within each subgroup were inferred based on the background model for the expected transcript count variability computed by the RaceID3 algorithm^36^. Using these distributions, a p-value for the observed difference in transcript counts between the two subgroups was calculated and multiple testing corrected by the Benjamini–Hochberg method. To assess the differential contribution of wildtype and mutant cells, we applied the Milo algorithm^48^, which models cell states as overlapping neighbourhoods based on k-nearest neighbour graphs as a basis for abundance testing. For visualization, neighbourhood graphs were mapped onto UMAP coordinates generated by the VarID algorithm. Each library of 96 cells was considered as one sample. Furthermore, the top 30 principal components from the VarID algorithm were used to build the k-nearest neighbour graph.

### Pathway analysis

Pathway analysis was carried out using algorithms provided by http://pantherdb.org/ (Ref.^31,32^) and https://reactome.org/ (Ref.^33^). Gene ontology analysis was carried out as described^47^.

### Statistics and reproducibility

All data analysis and plotting was performed using R version 4.1.3 or GraphPad Prism 9. For flow cytometry, BD FACSDiva v8.0.2. was used for data collection, and FlowJo 9.3.1 for flow cytometric analyses.

## Supplementary Information

Supplementary Information 1.

Supplementary Information 2.

Supplementary Information 3.

Supplementary Information 4.

Supplementary Information 5.

Supplementary Information 6.

Supplementary Information 7.

Supplementary Information 8.

Supplementary Information 9.

Supplementary Information 10.

Supplementary Information 11.

Supplementary Information 12.

## Data Availability

scRNA-seq data can be found at NCBI Gene Expression Omnibus (GEO) (GSE200756). RNA-seq data are deposited at NCBI Gene Expression Omnibus (GEO) (GSE214955).
