# Supplementary material for: Stage-specific and cell type-specific requirements of ikzf1 during haematopoietic differentiation in zebrafish
Source: Sci Rep. 2022 Dec 10;12:21401. doi: 10.1038/s41598-022-25978-6 (PMC9741631; doi:10.1038/s41598-022-25978-6)
Supplement: Supplementary file 12 — Supplementary Information 12. [file 41598_2022_25978_MOESM12_ESM.pdf]

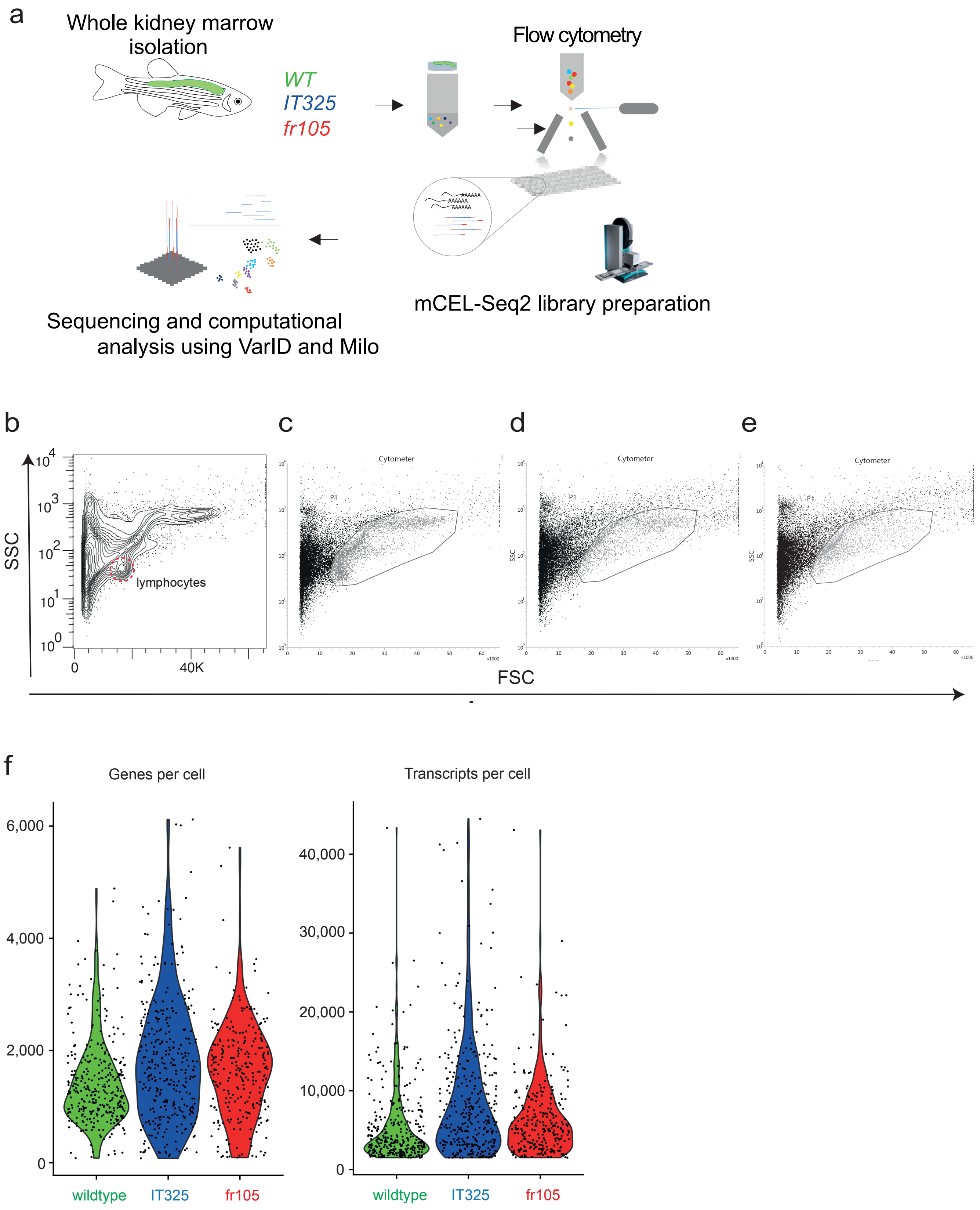
a
Whole kidney marrow isolation
WT
IT325
fr105
Flow cytometry
mCEL-Seq2 library preparation
Sequencing and computational analysis using VarID and Milo
b
c
d
e
SSC
lymphocytes
Cytometer
P1
FSC
f
Genes per cell
Transcripts per cell
wildtype
IT325
fr105

Supplementary Fig. 1. Single-cell transcriptome analysis. **a** Whole kidney marrow cell (WKM) suspensions were sorted as single cells by flow cytometry according to their light scatter characteristics (panel on the right; SSC, side scatter; FSC, forward scatter) and subjected to library preparation in microtitre plates. After lysis, they are subjected to cDNA synthesis and the mCEL-Seq2 protocol for library preparation. Finally, transcriptomes are sequenced by next generation sequencing and analyzed by VarID and Milo algorithms (see Methods). **b** Forward scatter (FSC) and side scatter (SSC) profiles of WKM cells isolated from wildtype fish at 3 months of age; the position of lymphocytes is indicated. **c**-**e**, Sort profiles for scRNA-seq analyses. c, wild-type, d, fr105 mutant, e, IT325 mutant. Gates are indicated. **f** Distribution of the number of genes and transcripts in cells of the indicated genotypes.
